# Supplementary figures and images for: Influence of CNTRENE® C100LM carbon nanotube material on the growth and regulation of Escherichia coli
Source: PeerJ. 2017 Aug 18;5:e3721. doi: 10.7717/peerj.3721 (PMC5564384; doi:10.7717/peerj.3721)

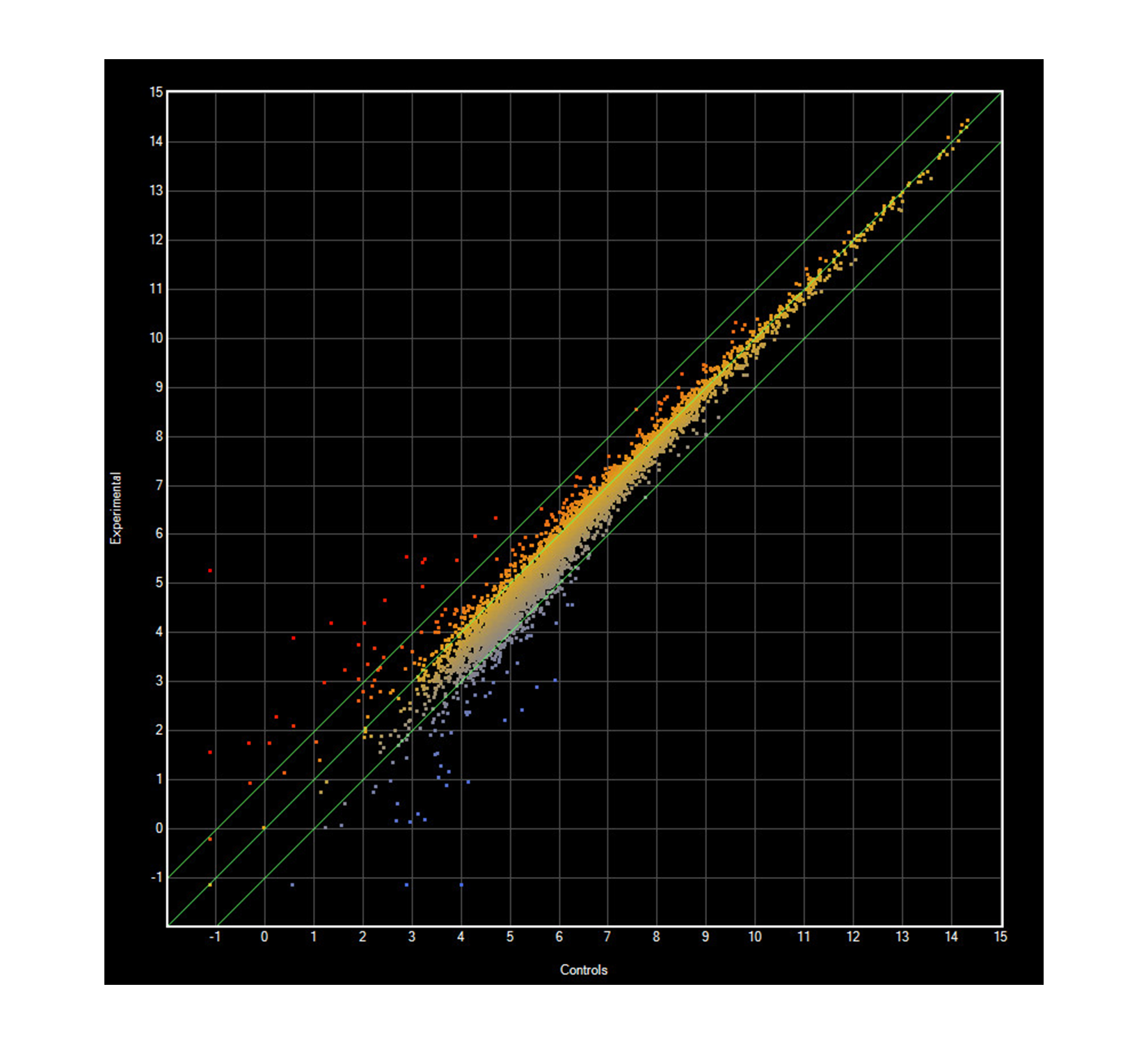

Supplement: Figure S1 — Mid-log phase E. coli K12 grown in M9 medium containing 1.05 µg/ml CNTs were compared to cultures without CNTRENE material exposure. Twenty-six genes were ≥2-fold upregulated in CNT exposed cells, while 160 genes were ≥2-fold downregulated in CNT exposed cell and 4,128 genes did not have differential expression (R2 = 0.9643). [file peerj-05-3721-s001.png]
